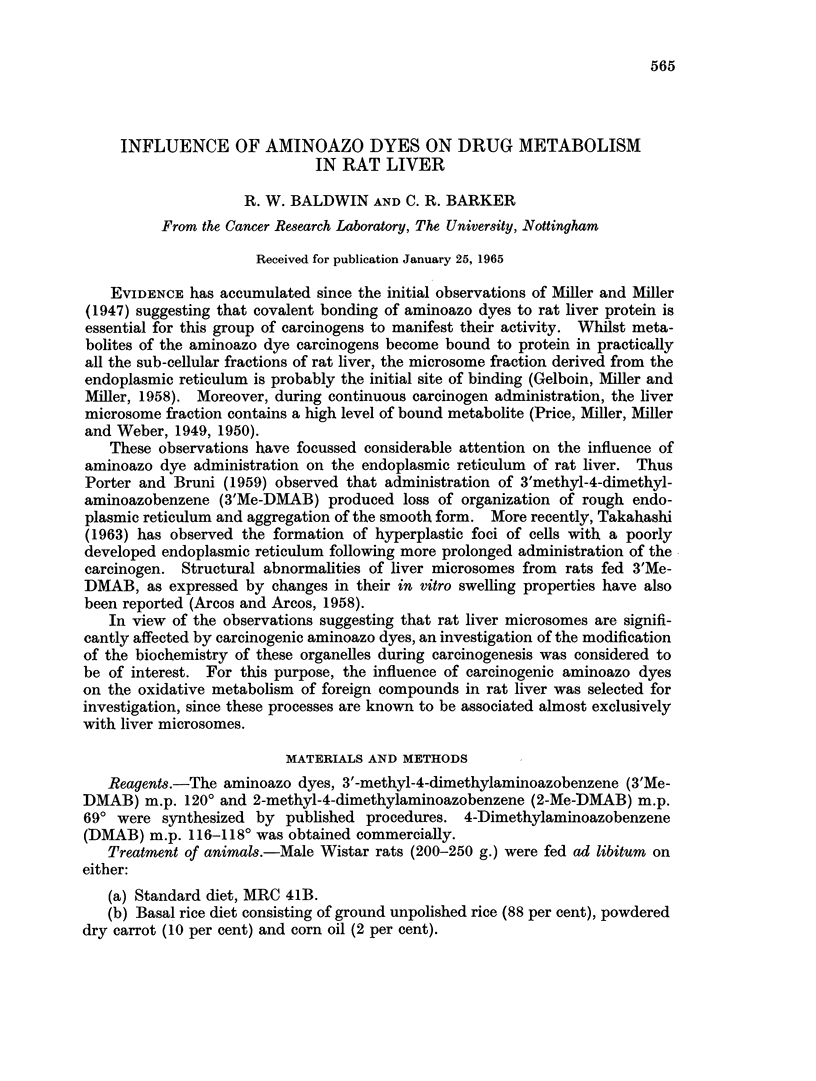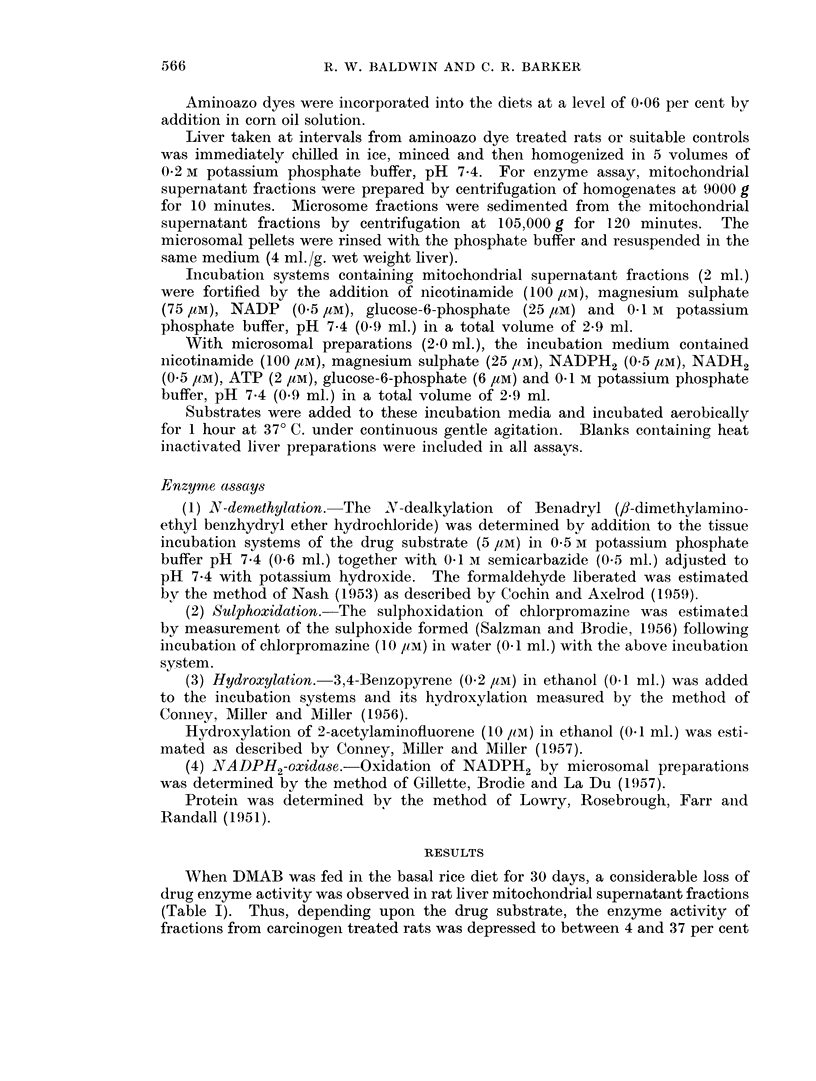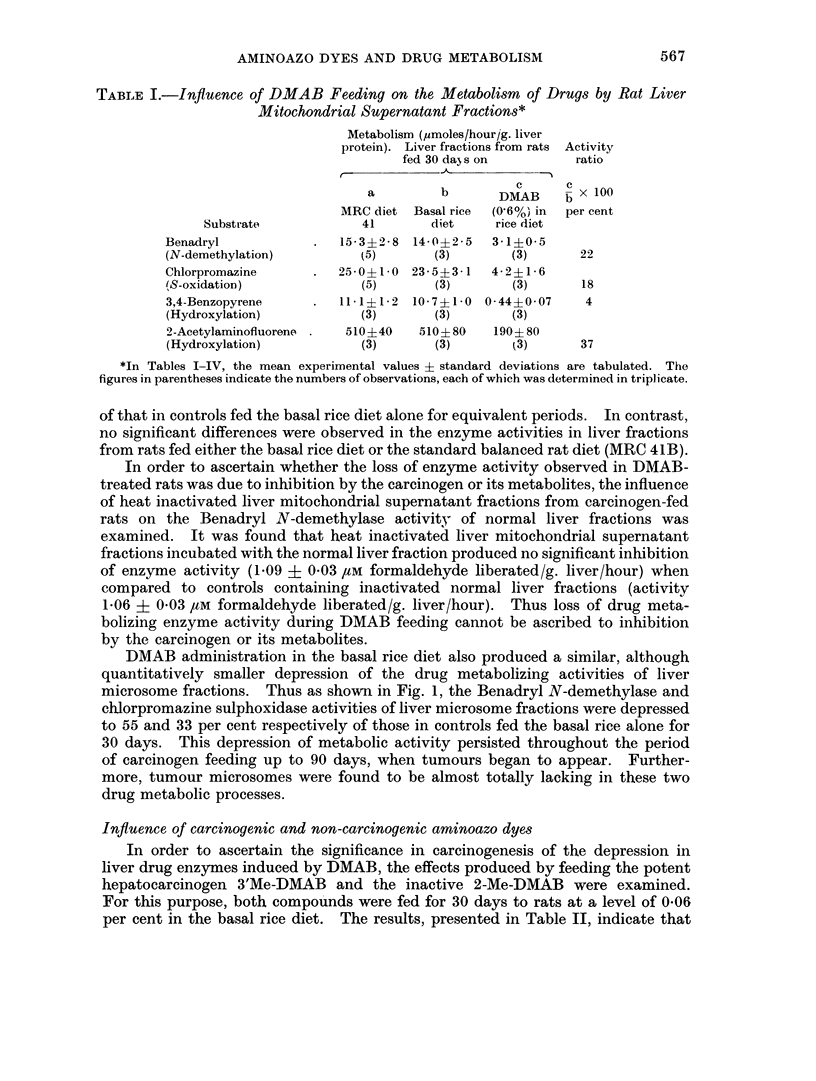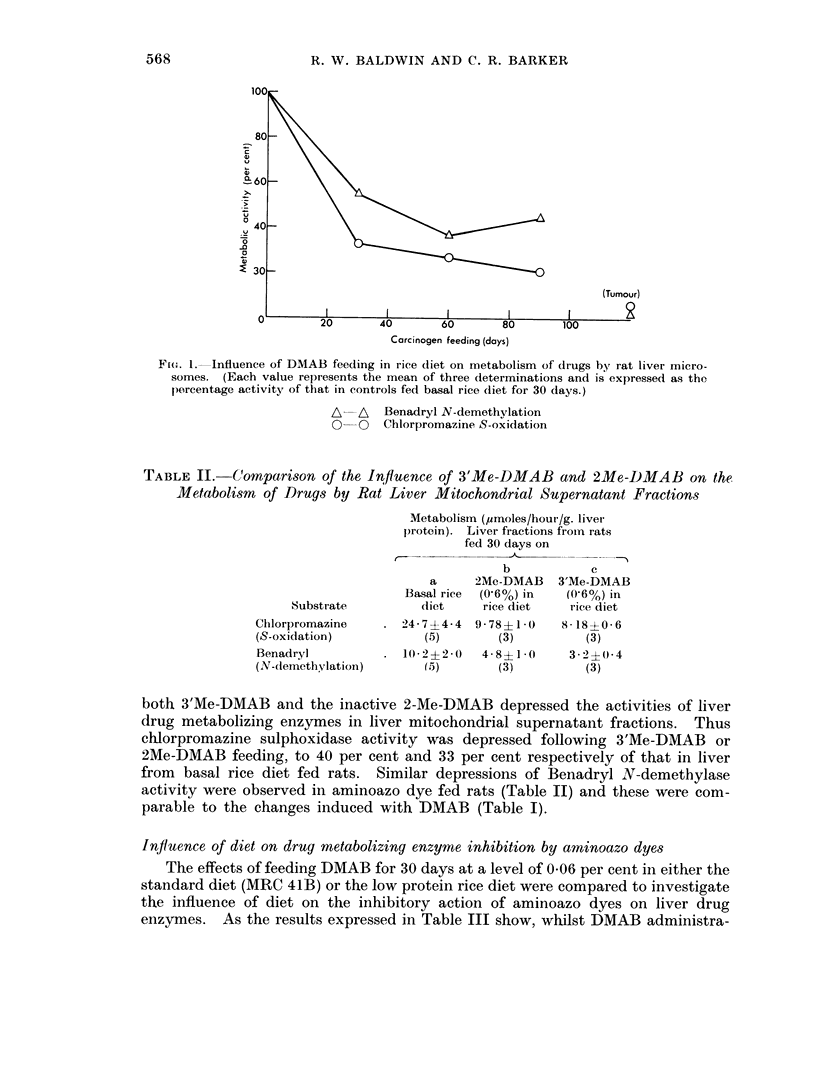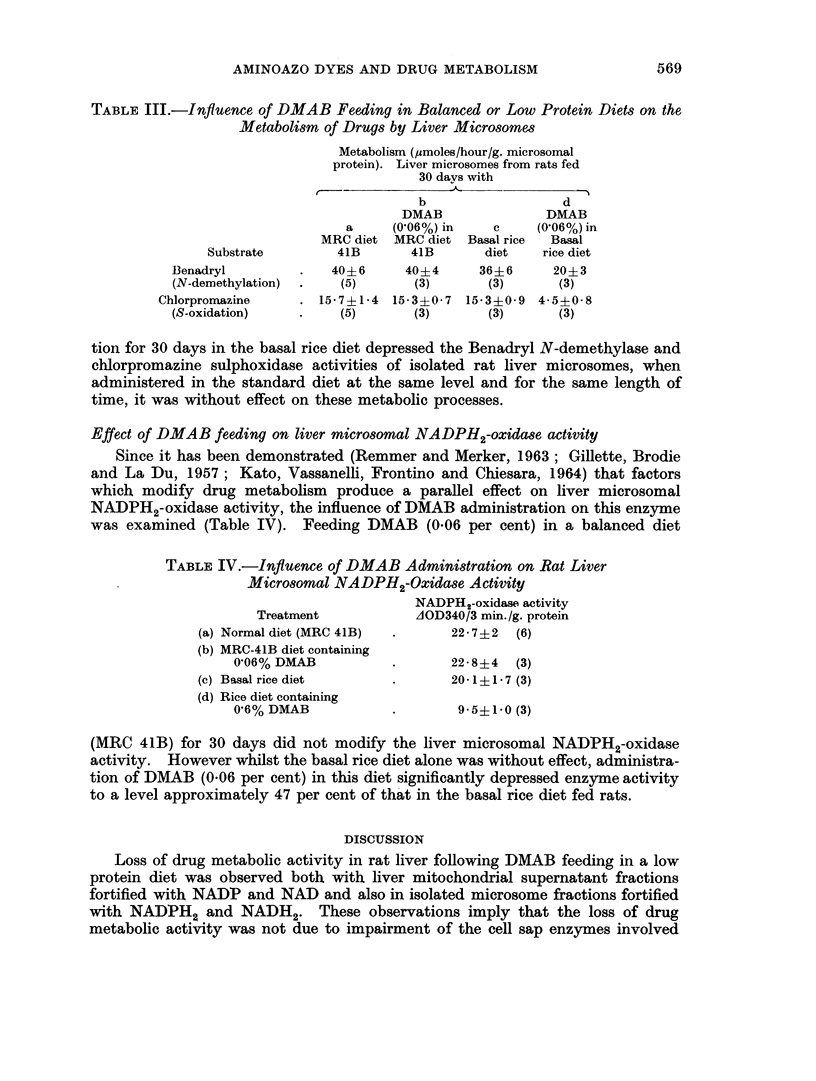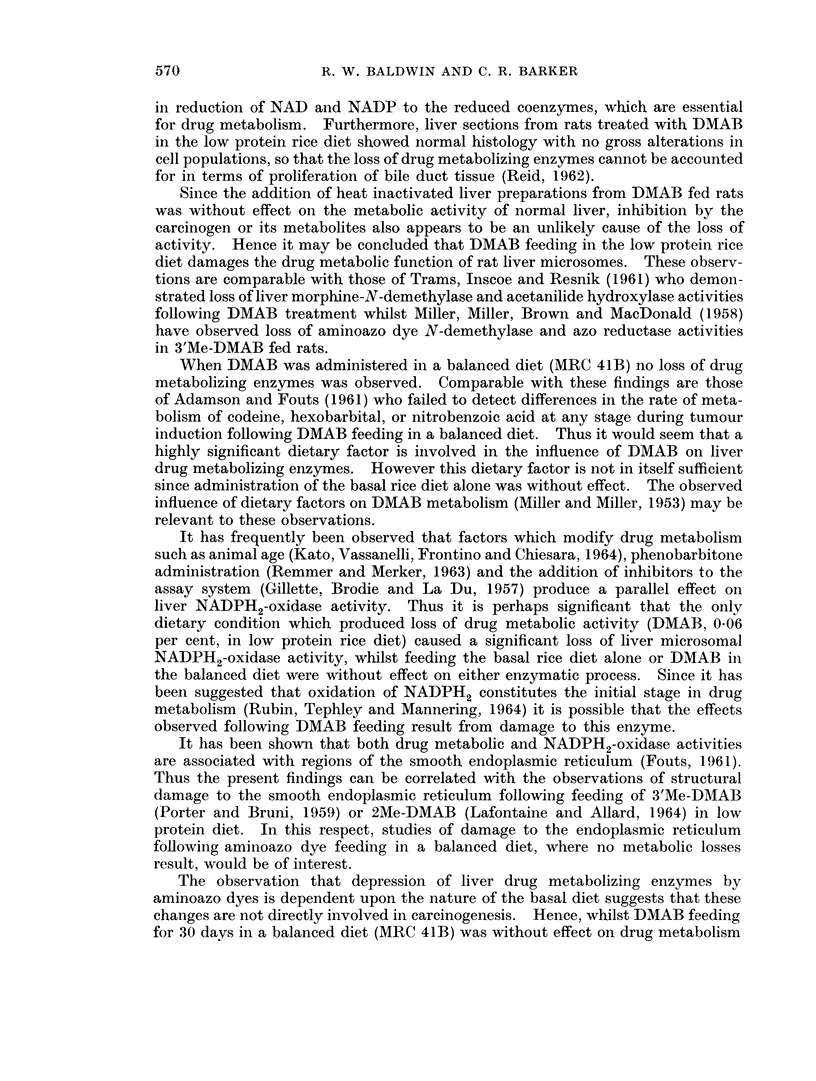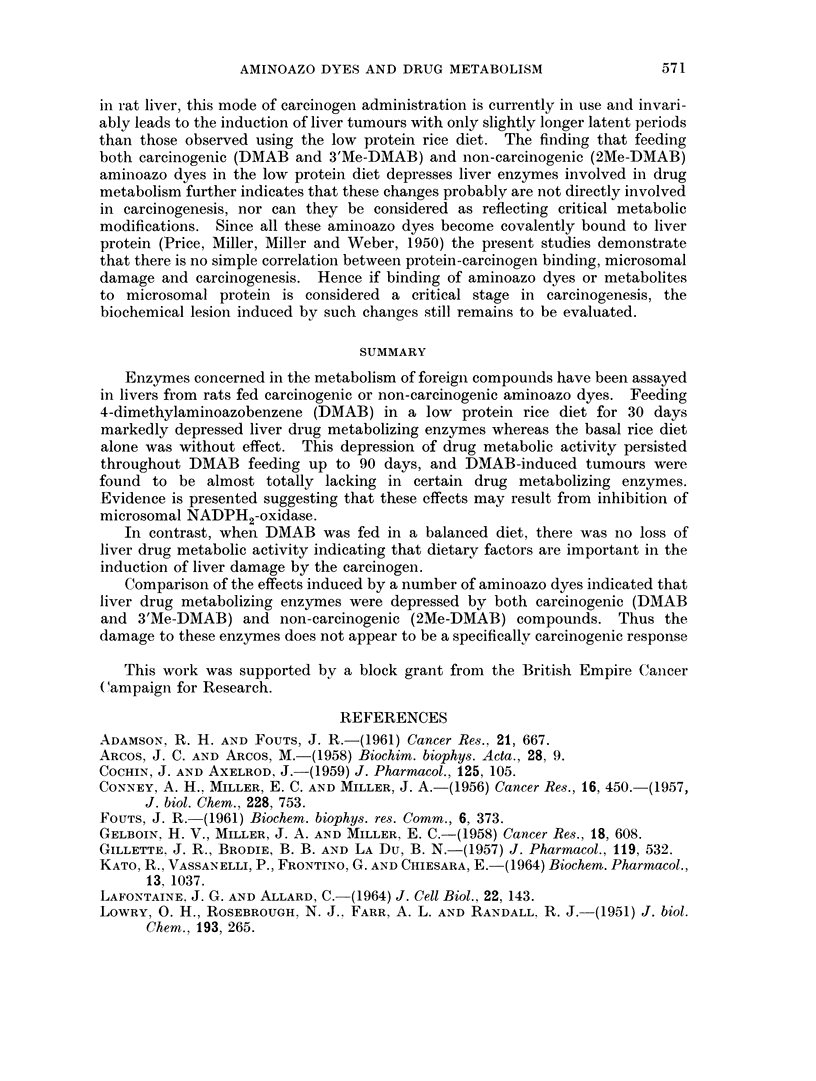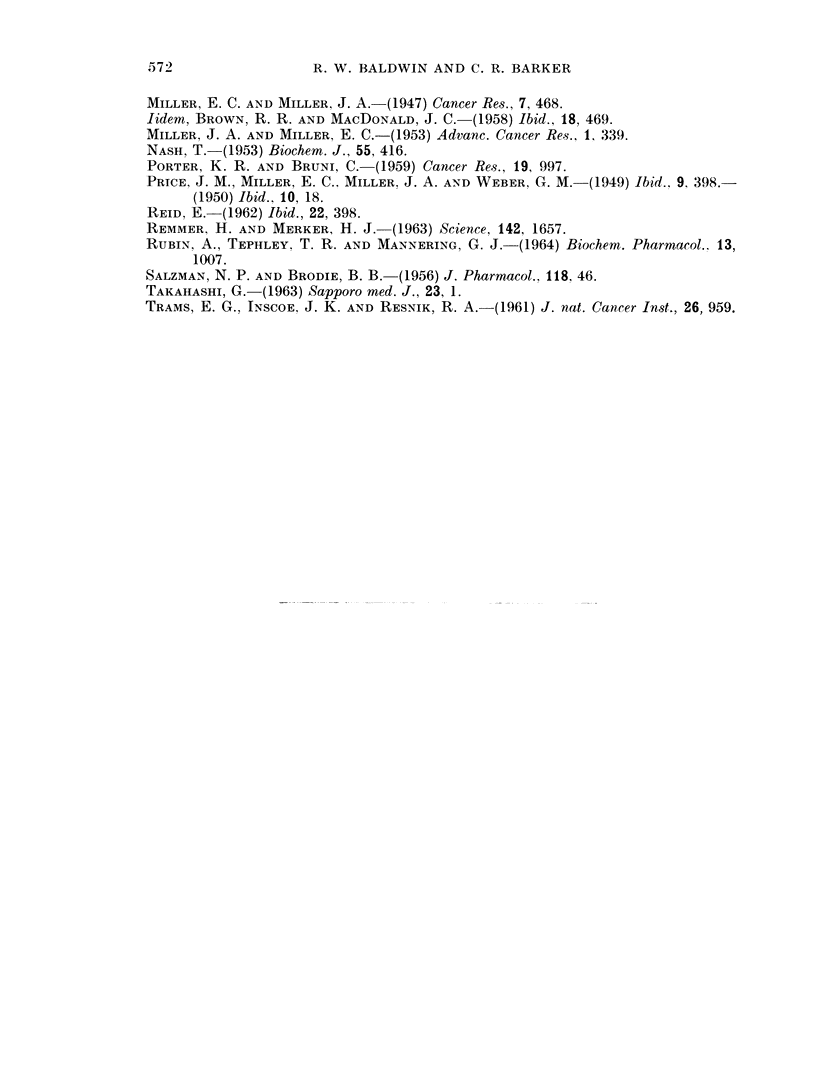# Influence of aminoazo dyes on drug metabolism in rat liver.

**DOI:** 10.1038/bjc.1965.70

**Published:** 1965-09

**Authors:** R. W. Baldwin, C. R. Barker


### INFLUENCE OF AMINOAZO DYES ON DRUG METABOLISM IN RAT LIVER

R. W. BALDWIN AND C. R. BARKER

*From the Cancer Research Laboratory, The University, Nottingham*

Received for publication January 25, 1965

EVIDENCE has accumulated since the initial observations of Miller and Miller (1947) suggesting that covalent bonding of aminoazo dyes to rat liver protein is essential for this group of carcinogens to manifest their activity. Whilst metabolites of the aminoazo dye carcinogens become bound to protein in practically all the sub-cellular fractions of rat liver, the microsome fraction derived from the endoplasmic reticulum is probably the initial site of binding (Gelboin, Miller and Miller, 1958). Moreover, during continuous carcinogen administration, the liver microsome fraction contains a high level of bound metabolite (Price, Miller, Miller and Weber, 1949, 1950).

These observations have focussed considerable attention on the influence of aminoazo dye administration on the endoplasmic reticulum of rat liver. Thus Porter and Bruni (1959) observed that administration of 3'methyl-4-dimethylaminoazobenzene (3'Me-DMAB) produced loss of organization of rough endoplasmic reticulum and aggregation of the smooth form. More recently, Takahashi (1963) has observed the formation of hyperplastic foci of cells with a poorly developed endoplasmic reticulum following more prolonged administration of the carcinogen. Structural abnormalities of liver microsomes from rats fed 3'Me-DMAB, as expressed by changes in their *in vitro* swelling properties have also been reported (Arcos and Arcos, 1958).

In view of the observations suggesting that rat liver microsomes are significantly affected by carcinogenic aminoazo dyes, an investigation of the modification of the biochemistry of these organelles during carcinogenesis was considered to be of interest. For this purpose, the influence of carcinogenic aminoazo dyes on the oxidative metabolism of foreign compounds in rat liver was selected for investigation, since these processes are known to be associated almost exclusively with liver microsomes.

### MATERIALS AND METHODS

*Reagents.*—The aminoazo dyes, 3'-methyl-4-dimethylaminoazobenzene (3'Me-DMAB) m.p. 120° and 2-methyl-4-dimethylaminoazobenzene (2-Me-DMAB) m.p. 69° were synthesized by published procedures. 4-Dimethylaminoazobenzene (DMAB) m.p. 116–118° was obtained commercially.

*Treatment of animals.*—Male Wistar rats (200–250 g.) were fed *ad libitum* on either:

(a) Standard diet, MRC 41B.

(b) Basal rice diet consisting of ground unpolished rice (88 per cent), powdered dry carrot (10 per cent) and corn oil (2 per cent).

Aminoazo dyes were incorporated into the diets at a level of 0·06 per cent by addition in corn oil solution.

Liver taken at intervals from aminoazo dye treated rats or suitable controls was immediately chilled in ice, minced and then homogenized in 5 volumes of 0·2 M potassium phosphate buffer, pH 7·4. For enzyme assay, mitochondrial supernatant fractions were prepared by centrifugation of homogenates at 9000 **g** for 10 minutes. Microsome fractions were sedimented from the mitochondrial supernatant fractions by centrifugation at 105,000 **g** for 120 minutes. The microsomal pellets were rinsed with the phosphate buffer and resuspended in the same medium (4 ml./g. wet weight liver).

Incubation systems containing mitochondrial supernatant fractions (2 ml.) were fortified by the addition of nicotinamide (100 $\mu$M), magnesium sulphate (75 $\mu$M), NADP (0·5 $\mu$M), glucose-6-phosphate (25 $\mu$M) and 0·1 M potassium phosphate buffer, pH 7·4 (0·9 ml.) in a total volume of 2·9 ml.

With microsomal preparations (2·0 ml.), the incubation medium contained nicotinamide (100 $\mu$M), magnesium sulphate (25 $\mu$M), $NADPH_2$ (0·5 $\mu$M), $NADH_2$ (0·5 $\mu$M), ATP (2 $\mu$M), glucose-6-phosphate (6 $\mu$M) and 0·1 M potassium phosphate buffer, pH 7·4 (0·9 ml.) in a total volume of 2·9 ml.

Substrates were added to these incubation media and incubated aerobically for 1 hour at 37° C. under continuous gentle agitation. Blanks containing heat inactivated liver preparations were included in all assays.

*Enzyme assays*

(1) *N-demethylation.*—The *N*-dealkylation of Benadryl ($\beta$-dimethylaminoethyl benzhydryl ether hydrochloride) was determined by addition to the tissue incubation systems of the drug substrate (5 $\mu$M) in 0·5 M potassium phosphate buffer pH 7·4 (0·6 ml.) together with 0·1 M semicarbazide (0·5 ml.) adjusted to pH 7·4 with potassium hydroxide. The formaldehyde liberated was estimated by the method of Nash (1953) as described by Cochin and Axelrod (1959).

(2) *Sulphoxidation.*—The sulphoxidation of chlorpromazine was estimated by measurement of the sulphoxide formed (Salzman and Brodie, 1956) following incubation of chlorpromazine (10 $\mu$M) in water (0·1 ml.) with the above incubation system.

(3) *Hydroxylation.*—3,4-Benzopyrene (0·2 $\mu$M) in ethanol (0·1 ml.) was added to the incubation systems and its hydroxylation measured by the method of Conney, Miller and Miller (1956).

Hydroxylation of 2-acetylaminofluorene (10 $\mu$M) in ethanol (0·1 ml.) was estimated as described by Conney, Miller and Miller (1957).

(4) *$NADPH_2$-oxidase.*—Oxidation of $NADPH_2$ by microsomal preparations was determined by the method of Gillette, Brodie and La Du (1957).

Protein was determined by the method of Lowry, Rosebrough, Farr and Randall (1951).

### RESULTS

When DMAB was fed in the basal rice diet for 30 days, a considerable loss of drug enzyme activity was observed in rat liver mitochondrial supernatant fractions (Table I). Thus, depending upon the drug substrate, the enzyme activity of fractions from carcinogen treated rats was depressed to between 4 and 37 per cent

TABLE I.—*Influence of DMAB Feeding on the Metabolism of Drugs by Rat Liver Mitochondrial Supernatant Fractions**

| Substrate | Metabolism ($\mu$moles/hour/g. liver protein). Liver fractions from rats fed 30 days on: a MRC diet 41 | b Basal rice diet | c DMAB (0·6%) in rice diet | Activity ratio $\frac{c}{b} \times 100$ per cent |
|---|---|---|---|---|
| Benadryl (*N*-demethylation) | 15·3±2·8 (5) | 14·0±2·5 (3) | 3·1±0·5 (3) | 22 |
| Chlorpromazine (*S*-oxidation) | 25·0±1·0 (5) | 23·5±3·1 (3) | 4·2±1·6 (3) | 18 |
| 3,4-Benzopyrene (Hydroxylation) | 11·1±1·2 (3) | 10·7±1·0 (3) | 0·44±0·07 (3) | 4 |
| 2-Acetylaminofluorene (Hydroxylation) | 510±40 (3) | 510±80 (3) | 190±80 (3) | 37 |

*In Tables I–IV, the mean experimental values ± standard deviations are tabulated. The figures in parentheses indicate the numbers of observations, each of which was determined in triplicate.

of that in controls fed the basal rice diet alone for equivalent periods. In contrast, no significant differences were observed in the enzyme activities in liver fractions from rats fed either the basal rice diet or the standard balanced rat diet (MRC 41B).

In order to ascertain whether the loss of enzyme activity observed in DMAB-treated rats was due to inhibition by the carcinogen or its metabolites, the influence of heat inactivated liver mitochondrial supernatant fractions from carcinogen-fed rats on the Benadryl *N*-demethylase activity of normal liver fractions was examined. It was found that heat inactivated liver mitochondrial supernatant fractions incubated with the normal liver fraction produced no significant inhibition of enzyme activity (1·09 ± 0·03 $\mu$M formaldehyde liberated/g. liver/hour) when compared to controls containing inactivated normal liver fractions (activity 1·06 ± 0·03 $\mu$M formaldehyde liberated/g. liver/hour). Thus loss of drug metabolizing enzyme activity during DMAB feeding cannot be ascribed to inhibition by the carcinogen or its metabolites.

DMAB administration in the basal rice diet also produced a similar, although quantitatively smaller depression of the drug metabolizing activities of liver microsome fractions. Thus as shown in Fig. 1, the Benadryl *N*-demethylase and chlorpromazine sulphoxidase activities of liver microsome fractions were depressed to 55 and 33 per cent respectively of those in controls fed the basal rice alone for 30 days. This depression of metabolic activity persisted throughout the period of carcinogen feeding up to 90 days, when tumours began to appear. Furthermore, tumour microsomes were found to be almost totally lacking in these two drug metabolic processes.

### *Influence of carcinogenic and non-carcinogenic aminoazo dyes*

In order to ascertain the significance in carcinogenesis of the depression in liver drug enzymes induced by DMAB, the effects produced by feeding the potent hepatocarcinogen 3′Me-DMAB and the inactive 2-Me-DMAB were examined. For this purpose, both compounds were fed for 30 days to rats at a level of 0·06 per cent in the basal rice diet. The results, presented in Table II, indicate that

FIG. 1.—Influence of DMAB feeding in rice diet on metabolism of drugs by rat liver microsomes. (Each value represents the mean of three determinations and is expressed as the percentage activity of that in controls fed basal rice diet for 30 days.)

△—△ Benadryl *N*-demethylation
○—○ Chlorpromazine *S*-oxidation

TABLE II.—*Comparison of the Influence of 3′Me-DMAB and 2Me-DMAB on the Metabolism of Drugs by Rat Liver Mitochondrial Supernatant Fractions*

| | Metabolism (μmoles/hour/g. liver protein). Liver fractions from rats fed 30 days on | | |
|---|---|---|---|
| Substrate | a Basal rice diet | b 2Me-DMAB (0·6%) in rice diet | c 3′Me-DMAB (0·6%) in rice diet |
| Chlorpromazine (*S*-oxidation) . | 24·7±4·4 (5) | 9·78±1·0 (3) | 8·18±0·6 (3) |
| Benadryl (*N*-demethylation) . | 10·2±2·0 (5) | 4·8±1·0 (3) | 3·2±0·4 (3) |

both 3′Me-DMAB and the inactive 2-Me-DMAB depressed the activities of liver drug metabolizing enzymes in liver mitochondrial supernatant fractions. Thus chlorpromazine sulphoxidase activity was depressed following 3′Me-DMAB or 2Me-DMAB feeding, to 40 per cent and 33 per cent respectively of that in liver from basal rice diet fed rats. Similar depressions of Benadryl *N*-demethylase activity were observed in aminoazo dye fed rats (Table II) and these were comparable to the changes induced with DMAB (Table I).

*Influence of diet on drug metabolizing enzyme inhibition by aminoazo dyes*

The effects of feeding DMAB for 30 days at a level of 0·06 per cent in either the standard diet (MRC 41B) or the low protein rice diet were compared to investigate the influence of diet on the inhibitory action of aminoazo dyes on liver drug enzymes. As the results expressed in Table III show, whilst DMAB administra-

TABLE III.—*Influence of DMAB Feeding in Balanced or Low Protein Diets on the Metabolism of Drugs by Liver Microsomes*

| Substrate | Metabolism (μmoles/hour/g. microsomal protein). Liver microsomes from rats fed 30 days with | | | |
|---|---|---|---|---|
| | a MRC diet 41B | b DMAB (0·06%) in MRC diet 41B | c Basal rice diet | d DMAB (0·06%) in Basal rice diet |
| Benadryl ($N$-demethylation) | 40±6 (5) | 40±4 (3) | 36±6 (3) | 20±3 (3) |
| Chlorpromazine ($S$-oxidation) | 15·7±1·4 (5) | 15·3±0·7 (3) | 15·3±0·9 (3) | 4·5±0·8 (3) |

tion for 30 days in the basal rice diet depressed the Benadryl $N$-demethylase and chlorpromazine sulphoxidase activities of isolated rat liver microsomes, when administered in the standard diet at the same level and for the same length of time, it was without effect on these metabolic processes.

*Effect of DMAB feeding on liver microsomal $NADPH_2$-oxidase activity*

Since it has been demonstrated (Remmer and Merker, 1963 ; Gillette, Brodie and La Du, 1957 ; Kato, Vassanelli, Frontino and Chiesara, 1964) that factors which modify drug metabolism produce a parallel effect on liver microsomal $NADPH_2$-oxidase activity, the influence of DMAB administration on this enzyme was examined (Table IV). Feeding DMAB (0·06 per cent) in a balanced diet

TABLE IV.—*Influence of DMAB Administration on Rat Liver Microsomal $NADPH_2$-Oxidase Activity*

| Treatment | $NADPH_2$-oxidase activity $\Delta$OD340/3 min./g. protein |
|---|---|
| (a) Normal diet (MRC 41B) | 22·7±2 (6) |
| (b) MRC-41B diet containing 0·06% DMAB | 22·8±4 (3) |
| (c) Basal rice diet | 20·1±1·7 (3) |
| (d) Rice diet containing 0·6% DMAB | 9·5±1·0 (3) |

(MRC 41B) for 30 days did not modify the liver microsomal $NADPH_2$-oxidase activity. However whilst the basal rice diet alone was without effect, administration of DMAB (0·06 per cent) in this diet significantly depressed enzyme activity to a level approximately 47 per cent of that in the basal rice diet fed rats.

### DISCUSSION

Loss of drug metabolic activity in rat liver following DMAB feeding in a low protein diet was observed both with liver mitochondrial supernatant fractions fortified with NADP and NAD and also in isolated microsome fractions fortified with $NADPH_2$ and $NADH_2$. These observations imply that the loss of drug metabolic activity was not due to impairment of the cell sap enzymes involved in reduction of NAD and NADP to the reduced coenzymes, which are essential for drug metabolism. Furthermore, liver sections from rats treated with DMAB in the low protein rice diet showed normal histology with no gross alterations in cell populations, so that the loss of drug metabolizing enzymes cannot be accounted for in terms of proliferation of bile duct tissue (Reid, 1962).

Since the addition of heat inactivated liver preparations from DMAB fed rats was without effect on the metabolic activity of normal liver, inhibition by the carcinogen or its metabolites also appears to be an unlikely cause of the loss of activity. Hence it may be concluded that DMAB feeding in the low protein rice diet damages the drug metabolic function of rat liver microsomes. These observtions are comparable with those of Trams, Inscoe and Resnik (1961) who demonstrated loss of liver morphine-*N*-demethylase and acetanilide hydroxylase activities following DMAB treatment whilst Miller, Miller, Brown and MacDonald (1958) have observed loss of aminoazo dye *N*-demethylase and azo reductase activities in 3′Me-DMAB fed rats.

When DMAB was administered in a balanced diet (MRC 41B) no loss of drug metabolizing enzymes was observed. Comparable with these findings are those of Adamson and Fouts (1961) who failed to detect differences in the rate of metabolism of codeine, hexobarbital, or nitrobenzoic acid at any stage during tumour induction following DMAB feeding in a balanced diet. Thus it would seem that a highly significant dietary factor is involved in the influence of DMAB on liver drug metabolizing enzymes. However this dietary factor is not in itself sufficient since administration of the basal rice diet alone was without effect. The observed influence of dietary factors on DMAB metabolism (Miller and Miller, 1953) may be relevant to these observations.

It has frequently been observed that factors which modify drug metabolism such as animal age (Kato, Vassanelli, Frontino and Chiesara, 1964), phenobarbitone administration (Remmer and Merker, 1963) and the addition of inhibitors to the assay system (Gillette, Brodie and La Du, 1957) produce a parallel effect on liver $NADPH_2$-oxidase activity. Thus it is perhaps significant that the only dietary condition which produced loss of drug metabolic activity (DMAB, 0·06 per cent, in low protein rice diet) caused a significant loss of liver microsomal $NADPH_2$-oxidase activity, whilst feeding the basal rice diet alone or DMAB in the balanced diet were without effect on either enzymatic process. Since it has been suggested that oxidation of $NADPH_2$ constitutes the initial stage in drug metabolism (Rubin, Tephley and Mannering, 1964) it is possible that the effects observed following DMAB feeding result from damage to this enzyme.

It has been shown that both drug metabolic and $NADPH_2$-oxidase activities are associated with regions of the smooth endoplasmic reticulum (Fouts, 1961). Thus the present findings can be correlated with the observations of structural damage to the smooth endoplasmic reticulum following feeding of 3′Me-DMAB (Porter and Bruni, 1959) or 2Me-DMAB (Lafontaine and Allard, 1964) in low protein diet. In this respect, studies of damage to the endoplasmic reticulum following aminoazo dye feeding in a balanced diet, where no metabolic losses result, would be of interest.

The observation that depression of liver drug metabolizing enzymes by aminoazo dyes is dependent upon the nature of the basal diet suggests that these changes are not directly involved in carcinogenesis. Hence, whilst DMAB feeding for 30 days in a balanced diet (MRC 41B) was without effect on drug metabolism in rat liver, this mode of carcinogen administration is currently in use and invariably leads to the induction of liver tumours with only slightly longer latent periods than those observed using the low protein rice diet. The finding that feeding both carcinogenic (DMAB and 3'Me-DMAB) and non-carcinogenic (2Me-DMAB) aminoazo dyes in the low protein diet depresses liver enzymes involved in drug metabolism further indicates that these changes probably are not directly involved in carcinogenesis, nor can they be considered as reflecting critical metabolic modifications. Since all these aminoazo dyes become covalently bound to liver protein (Price, Miller, Miller and Weber, 1950) the present studies demonstrate that there is no simple correlation between protein-carcinogen binding, microsomal damage and carcinogenesis. Hence if binding of aminoazo dyes or metabolites to microsomal protein is considered a critical stage in carcinogenesis, the biochemical lesion induced by such changes still remains to be evaluated.

### SUMMARY

Enzymes concerned in the metabolism of foreign compounds have been assayed in livers from rats fed carcinogenic or non-carcinogenic aminoazo dyes. Feeding 4-dimethylaminoazobenzene (DMAB) in a low protein rice diet for 30 days markedly depressed liver drug metabolizing enzymes whereas the basal rice diet alone was without effect. This depression of drug metabolic activity persisted throughout DMAB feeding up to 90 days, and DMAB-induced tumours were found to be almost totally lacking in certain drug metabolizing enzymes. Evidence is presented suggesting that these effects may result from inhibition of microsomal $NADPH_2$-oxidase.

In contrast, when DMAB was fed in a balanced diet, there was no loss of liver drug metabolic activity indicating that dietary factors are important in the induction of liver damage by the carcinogen.

Comparison of the effects induced by a number of aminoazo dyes indicated that liver drug metabolizing enzymes were depressed by both carcinogenic (DMAB and 3'Me-DMAB) and non-carcinogenic (2Me-DMAB) compounds. Thus the damage to these enzymes does not appear to be a specifically carcinogenic response

This work was supported by a block grant from the British Empire Cancer Campaign for Research.

### REFERENCES

ADAMSON, R. H. AND FOUTS, J. R.—(1961) *Cancer Res.*, **21**, 667.
ARCOS, J. C. AND ARCOS, M.—(1958) *Biochim. biophys. Acta.*, **28**, 9.
COCHIN, J. AND AXELROD, J.—(1959) *J. Pharmacol.*, **125**, 105.
CONNEY, A. H., MILLER, E. C. AND MILLER, J. A.—(1956) *Cancer Res.*, **16**, 450.—(1957, *J. biol. Chem.*, **228**, 753.
FOUTS, J. R.—(1961) *Biochem. biophys. res. Comm.*, **6**, 373.
GELBOIN, H. V., MILLER, J. A. AND MILLER, E. C.—(1958) *Cancer Res.*, **18**, 608.
GILLETTE, J. R., BRODIE, B. B. AND LA DU, B. N.—(1957) *J. Pharmacol.*, **119**, 532.
KATO, R., VASSANELLI, P., FRONTINO, G. AND CHIESARA, E.—(1964) *Biochem. Pharmacol.*, **13**, 1037.
LAFONTAINE, J. G. AND ALLARD, C.—(1964) *J. Cell Biol.*, **22**, 143.
LOWRY, O. H., ROSEBROUGH, N. J., FARR, A. L. AND RANDALL, R. J.—(1951) *J. biol. Chem.*, **193**, 265.

MILLER, E. C. AND MILLER, J. A.—(1947) *Cancer Res.*, **7**, 468.
*Iidem*, BROWN, R. R. AND MACDONALD, J. C.—(1958) *Ibid.*, **18**, 469.
MILLER, J. A. AND MILLER, E. C.—(1953) *Advanc. Cancer Res.*, **1**, 339.
NASH, T.—(1953) *Biochem. J.*, **55**, 416.
PORTER, K. R. AND BRUNI, C.—(1959) *Cancer Res.*, **19**, 997.
PRICE, J. M., MILLER, E. C., MILLER, J. A. AND WEBER, G. M.—(1949) *Ibid.*, **9**, 398.—(1950) *Ibid.*, **10**, 18.
REID, E.—(1962) *Ibid.*, **22**, 398.
REMMER, H. AND MERKER, H. J.—(1963) *Science*, **142**, 1657.
RUBIN, A., TEPHLEY, T. R. AND MANNERING, G. J.—(1964) *Biochem. Pharmacol.*, **13**, 1007.
SALZMAN, N. P. AND BRODIE, B. B.—(1956) *J. Pharmacol.*, **118**, 46.
TAKAHASHI, G.—(1963) *Sapporo med. J.*, **23**, 1.
TRAMS, E. G., INSCOE, J. K. AND RESNIK, R. A.—(1961) *J. nat. Cancer Inst.*, **26**, 959.